# Antioxidant Capacity and Phenolic Profile of Edible Carob Pods (*Ceratonia siliqua* L.)

**DOI:** 10.3390/foods15183197

**Published:** 2026-09-10

**Authors:** Stamatia Angeliki Kleftaki, Thalia Tsiaka, Georgios Bekiaris, Eleni Papapanagi, Charalampia Amerikanou, Sotirios-Spyridon Vamvakas, Aristea Gioxari, Panagiotis Zoumpoulakis, Andriana C. Kaliora

**Affiliations:** 1 Department of Nutrition and Dietetics, School of Health Science and Education, Harokopio University of Athens, 70 El. Venizelou Ave., 17676 Athens, Greececamer@hua.gr (C.A.); 2 Laboratory of Chemistry, Analysis & Design of Food Processes, Department of Food Science and Technology, University of West Attica, Agiou Spyridonos, 12243 Athens, Greece; 3 Laboratory of Food Microbiology and Biotechnology, Department of Food Science and Human Nutrition, Agricultural University of Athens, 11855 Athens, Greece; g.bekiaris@aua.gr; 4 Department of Nutritional Science and Dietetics, University of the Peloponnese, 24100 Kalamata, Greece; nds22078@go.uop.gr (E.P.); a.gioxari@go.uop.gr (A.G.)

**Keywords:** carob, *Ceratonia siliqua*, phenolic compounds, antioxidant capacity, LC-MS, nutraceuticals

## Abstract

Our study aimed, for the first time, to comparatively characterize edible carob powder from the Imera and Platses cultivars in terms of phenolic composition and antioxidant capacity, providing the first comprehensive characterization of the underexplored Platses cultivar using an integrated multi-analytical approach. FT-IR fingerprinting, GC-MS volatile profiling and LC-MS phenolic analysis were performed, while total phenolic content and antioxidant capacity were evaluated using Folin–Ciocalteu, FRAP, DPPH, ABTS and TSO assays. FT-IR indicated higher absorption in regions related to α- and β-glucans in Platses, while GC-MS identified 22 volatile compounds, with isobutyric acid being the dominant volatile in both cultivars. LC-MS revealed distinct phenolic profiles, with Imera exhibiting higher relative abundances of flavonoids, galloylated and flavanol derivatives, whereas Platses showed higher relative abundances of gallic acid, ferulic acid and kaempferol. Imera also exhibited significantly higher total phenolic and flavanol contents and antioxidant capacity (FRAP, DPPH, and ABTS; *p* < 0.05), while TSO did not differ between cultivars. Strong positive correlations were observed between phenolic content and antioxidant activity. These findings support the exploitation of Cretan carob cultivars as natural sources of bioactive compounds for functional food and nutraceutical applications.

## 1. Introduction

The carob tree (*Ceratonia siliqua* L.) belongs to the Fabaceae family and has been widely cultivated in the Mediterranean basin offering significant economic and social development to European and Middle East countries, such as Spain, Portugal, Greece, Egypt and Morocco [1]. Over the last few years, carob cultivation has regained attention, as it increases resilience and supports sustainability of agriculture systems, mainly due to its resistance to drought, salinity, and other biotic and abiotic stresses [2,3].

Carob pods are considered as an important source of sugar dietary fibers, mainly insoluble, and other bioactive compounds, such as polyphenols and d-pinitol, while their consumption has been associated with several health benefits, such as antioxidant, anti-inflammatory, cardioprotective and others [4]. In vitro and in vivo studies have shown that carob pods exhibit significant radical scavenging activity, mainly due to their high flavonoid and polyphenol content, with evidence of reducing oxidative stress in animal models [5,6,7,8]. Τhe volatile composition of carob includes acids, esters, aldehydes, ketones, alcohols, and furans, which contribute to its characteristic aroma [9,10], with roasting determining the sensory and aromatic properties of carob products [11].

Phytochemical characterization of carob extracts has contributed to the association of carob pods’ bioactivities with specific antioxidant compounds, such as phenolic compounds. Several phytochemical profiling analyses have been conducted in carob pods for the identification of their main phenolic components, mainly applying high-performance liquid chromatography (HPLC) [5,12,13]. Recently, liquid chromatography coupled with high-resolution time-of-flight mass spectrometry (LC-TOF MS) has been considered the most sensitive and appropriate analytical technique for the evaluation of phenolic composition due to its high mass resolution and accuracy [14,15,16,17], while Fourier-Transform Infrared spectroscopy (FTIR) is a fast, reliable and non-destructive method to achieve an overall molecular fingerprint. In particular, FTIR offers a chemical insight into the examined sample by providing a unique spectrum [18,19] of fingerprint-like functional groups. It has been previously applied in carob pods extracts confirming the potential of a fast and reliable screening tool on the basis of antioxidant activity and phenolic content in carob extracts [20,21].

Greece is one of the key producers of carob with Crete hosting Europe’s largest natural carob tree habitat [22]. Several cultivars have been recorded in Crete with the most typical being the Imera (or Hemere) cultivar, whose genome was recently sequenced, assembled and annotated for the first time for C. siliqua [23]. Extracts from the Imera cultivar were recently investigated in terms of sugar content, fatty acid composition and biological activities, showing promising antioxidant activity [23]. However, Platses, another traditionally classified cultivar found in Crete [22], remains unexplored in terms of its phytochemical composition and antioxidant potential. Comparative studies of these cultivars using advanced analytical approaches remain limited, yet they are essential for elucidating cultivar-dependent differences that may influence the nutritional and health-promoting properties of carob-derived products.

Hence, the present study aimed, for the first time, to comparatively characterize edible carob powder from the Imera and Platses cultivars through LC-TOF-MS-based phenolic profiling, together with the evaluation of their antioxidant capacity, FT-IR fingerprinting, volatile composition, and nutritional characteristics. The novelty of this work lies in the first comprehensive characterization of the underexplored Platses cultivar using an integrated multi-analytical approach.

## 2. Materials and Methods

### 2.1. Chemicals and Plant Materials

The plant materials used in this study were kindly provided by local producers from the island of Crete, Greece. The trees were botanically identified by Professor Kalantidis, Department of Biology, University of Crete, Greece. After the mechanical separation of the seeds from the pods, samples were roasted (140 °C, 40 min) and finally ground into fine powders. These roasting conditions were selected to achieve a medium-flavor carob powder, balancing the development of roasted characteristics while avoiding excessively burnt profiles, and are commonly applied in Crete for the production of edible carob powder. Identical roasting conditions were applied to both cultivars to allow their direct comparison under the same thermal processing conditions. Therefore, the observed compositional differences should be interpreted as the combined effect of cultivar-specific characteristics and differential responses to the applied thermal processing conditions, rather than exclusively as intrinsic differences between the two cultivars.

Methanol and water were acquired from PanReac AppliChem ITW Reagents (Darmstadt, Germany), while formic acid and acetonitrile were obtained by Carlo Erba (Milan, Italy) and Fisher Scientific (Leicestershire, UK), respectively. All solvents were LC-MS grade. In all cases, samples were analyzed in triplicate. All reagents and chemicals used in this study were of analytical grade and were purchased from Sigma-Aldrich (St. Louis, MO, USA).

### 2.2. Proximate Composition Analysis

Moisture was measured by air-oven drying, while ash content was determined by the dry ashing technique. Crude protein content of the powder was determined using Kjeldahl method and the converting factor 6.25 (Selecta, S.A., Barcelona, Spain). The lipid content was measured by the colorimetric sulfo-phospho-vanillin reaction, employing commercial sunflower oil as a lipid standard [24,25]. Gross energy content was determined by an adiabatic bomb calorimeter (IKA C4000, Analysentechnik, Heitersheim, Germany). Crude fiber was determined by Weende’s method using a Dosi Fiber apparatus (Selecta, S.A., Barcelona, Spain). The method is based on the solubilization (digestion) of non-cellulosic compounds by a sulfuric acid and potassium hydroxide solution. Crude fiber is the loss on ignition of the dried residue remaining after digestion of the sample, determined gravimetrically. Carbohydrate content was calculated as follows:

Carbohydrates=(amount of total sample)−(Moisture)−(Protein)−(Fat)−(ash)

All measurements were conducted in triplicate.

### 2.3. Preparation of Extracts for Spectrophotometric and LC-TOF MS Analyses

Precisely 1.0 g of each carob powder was weighed and subsequently 10.0 mL of methanol–water mixture (70:30% *v*/*v*) were added as extraction solvent. The extraction tubes were stirred at room temperature for 18 h. Then, the extracts were centrifuged at 3500 rpm for 15 min and the supernatants were stored at −20 °C until further analyses. For LC-MS experiments, 1.0 mL of each extract was freeze-dried and the obtained dry residues were re-constituted at 1.0 mL water–acetonitrile (90:10% *v*/*v*) containing 0.1% *v*/*v* formic acid. Prior to LC-MS analysis, all samples were filtered using a Chromafil Xtra PET filter 45/13 (Macherey-Nagel, Düren Germany). All measurements below were conducted in triplicate.

### 2.4. Total Phenolic Content, Total Flavanol Content and Antioxidant Activity Assays

Total phenolics of the aqueous methanolic extract was performed using the Folin–Ciocalteau method and by measuring the absorbance at 750 nm. Gallic acid was used as calibration standard and results were expressed as mg gallic acid equivalents (mg GAE) per g of carob powder, according to the method of Tsiaka et al. [26].

Total flavanols were measured with the p-dimethylaminocinnamaldehyde (DMACA) assay [17] and the results were expressed as μg catechin equivalents per g of carob powder.

The antioxidant and antiradical capacity were evaluated with ferric reducing antioxidant power (FRAP) and 2,2-diphenyl-1-picrylhydrazyl (DPPH) and (2,2′-azino-bis(3-ethylbenzothiazoline-6-sulfonic acid) (ABTS•+) assays, respectively. Results of FRAP and ABTS•+ were expressed as mg Fe (II) equivalents per g of carob powder, and mg Trolox equivalents (TE) per g of carob powder, respectively, as described in the work of Tsiaka et al. [25]. Hydrogen-donating capacity was determined with the DPPH radical scavenging assay using a commercial colorimetric DPPH antioxidant assay kit (Abcam, Cambridge, MA, USA) according to the manufacturer’s instructions and was expressed as mg TE per g of carob powder.

Copper-induced lipid oxidation inhibition of human serum solubilized in phosphate-buffered saline (PBS) was measured using the total serum oxidizability (TSO) assay [27]. The kinetics of oxidation in diluted serum were performed by measuring the absorbance of lipid oxidation products. CuSO4 was added in 20 µL of serum to a final concentration of 10^−5^ M in PBS. Copper-induced oxidation of lipids leads to conjugated dienic hydroperoxides formation that absorb at 245 nm. The kinetics were analyzed in terms of lag time prior to oxidation and were expressed in seconds, relative to the control. All analyses were performed in triplicate.

All measurements were performed using a microplate spectrophotometer (PowerWave XS2, BioTek Instruments, Winooski, VT, USA).

### 2.5. LC-MS/MS Analysis of Carob Extracts

The phenolic profiling of carob samples was conducted using an ekspert™ nanoLC 425 chromatograph (Eksigent, Dublin, CA, USA) combined with TripleTOF^®^ 6600+ mass spectrometry detector with DuoSpray ion source (Sciex, Framingham, MA, USA). The elution of phenolic compounds was performed by a reversed-phase chromatography column Luna C-18(2) 100 Å (Phenomenex, Torrance, CA, USA) with an internal diameter of 150 mm × 0.3 mm and a particle size of 3 μm at microflow rate of 10 μL/min. The mobile phase included water −0.1% *v*/*v* formic acid (Solvent A) and acetonitrile −0.1% *v*/*v* formic acid (Solvent B) and the gradient was programmed over 12 min. MS and MS/MS acquisition were carried out using electrospray ionization (ESI) at negative ionization mode, while Information Dependent Acquisition (IDA) mode was implemented for MS/MS fragmentation. The conditions of applied LC-MS method are thoroughly described in the work of Amerikanou et al. [28]. LC-MS analysis and data processing was performed using Analyst TF version 1.8 and Sciex OS software (Sciex, Framingham, MA, USA).

### 2.6. FTIR Analysis

The ATR-FTIR spectra of carob samples were recorded by a Perkin Elmer Spectrum-Two spectrometer equipped with a Diamond ATR compartment (Perkin Elmer, Hopkinton, MA, USA) using the provided by the manufacturer Spectrum 10 software (v.10.5.1.581). For each samples’ spectrum, 32 scans in the mid-infrared region (4000 and 400 cm^−1^) at a resolution of 4 cm^−1^ were averaged and ATR-corrected using a refractive index for diamond crystal of 1.5, in order to be comparable to the available spectral libraries. In addition to crystal correction, the spectra were smoothed by Savitzky–Golay algorithm (smoothing window of 7 cm^−1^), linearly baseline corrected and normalized by the mean using The Unscrambler X v.10.5 software, Version 10.5 (CAMO software, Oslo, Norway).

### 2.7. Gas Chromatography Mass Spectrometry (GC-MS) Analysis of Volatile Compounds

Carob powder (1 g, dry weight) was extracted with 15 mL of 70% (*v*/*v*) ethanol using an ultrasonic bath operated at ambient temperature. Ultrasonic assisted extraction was performed in two consecutive 10 min cycles to disrupt the sugar- and fiber-rich carob matrix, enhance solvent penetration, and facilitate the transfer of volatile compounds into the solvent phase before subsequent concentration of the extract. Following sonication, the mixture was incubated at room temperature for 4 h to allow sufficient extraction equilibrium. The suspension was subsequently centrifuged at 4500× *g* for 5 min, and the resulting supernatant was collected.

The crude ethanolic extract was subjected to liquid–liquid extraction to isolate nonpolar components. Initially, the extract was mixed with 15 mL of hexane, vortexed thoroughly, and allowed to separate into two distinct phases. The organic layer was collected, and a second extraction was performed using an additional 10 mL of hexane to maximize recovery of hydrophobic constituents. The combined hexane fractions were dried over anhydrous Na_2_SO_4_ and filtered through a 0.22 μm membrane to remove residual moisture and particulate matter.

The clarified organic phase was concentrated under mild conditions, avoiding elevated temperatures to prevent thermal degradation, until a final volume of approximately 1 mL was obtained. The concentrated extract was transferred to amber vials and stored at 4 °C, until further analysis.

Volatile compounds were analyzed using a GC-MS system (Shimadzu Corporation, Kyoto, Japan) equipped with a MEGA 5HT capillary column (MEGA S.r.l., Legnano, Italy). Samples were injected in splitless mode with a sampling time of 0.5 min, using an injection volume of 1 μL and an injector temperature of 350 °C. Helium was used as the carrier gas at a constant flow rate of 0.9 mL/min. The oven temperature program was set as follows: initial temperature 30 °C (held for 1.50 min), increased to 230 °C at 5 °C/min with a 3 min hold, and subsequently raised to 320 °C at 20 °C/min with a final hold of 3 min. The mass spectrometer operated with an ion source temperature of 230 °C and an interface temperature of 280 °C. Electron ionization at 70 eV was applied, and mass spectra were acquired in full scan mode over an *m*/*z* range of 40–500. Compound identification was performed based on mass spectral matching.

### 2.8. Statistical Analysis

Quantitative variables are expressed as mean values (standard deviation) and are compared between the two cultivars via Independent Samples *t* Test. Pearson’s correlation was used to evaluate correlations between phenolic content and the antioxidant and antiradical activities of the two cultivars. Statistical significance was set at *p* ≤ 0.05 and analyses were conducted using SPSS statistical software, Version 23.0 (IBM, SPSS Inc., Chicago, IL, USA).

## 3. Results

### 3.1. Proximate Analysis

The proximate composition of the carobs is presented in Table 1 to provide a general nutritional characterization of the samples. As shown, both cultivars exhibited similar macronutrient profiles, with carbohydrates being the predominant component, while minor variations were observed in protein and fat content.

### 3.2. Evaluation of Total Phenolic Content (TPC), Total Flavanols, Antioxidant and Antiradical Activity of Carob Cultivars Imera and Platses

The aqueous methanolic extracts of Imera and Platses powders were evaluated for their phenolic and flavanol content and their antioxidant and antiradical capacity (Table 2). The antioxidant potential of the Imera cultivar was significantly higher (*p*-value < 0.05) compared with Platses, as reflected by all tested assays. Specifically, Imera exhibited significantly greater (*p*-value < 0.05) total phenolic and flavanol content, as well as enhanced radical scavenging activity (DPPH and ABTS), FRAP, indicating differences in phenolic composition between the two cultivars (Table 2).

Pearson’s correlation test was conducted to assess the degree of correlation between the phenolic content of the two carob powders and their antioxidant and antiradical activities. High values of Pearson’s coefficients (namely, *p* ≤ 0.05, R > 0.8) implied a very strong positive correlation among TPC and reducing power activity (R = 0.878), TPC and antiradical activity (R = 0.933 for ABTS•+ and R = 0.934 for DPPH), as well as between reducing power (FRAP) and radical scavenging activity (R = 0.987 for ABTS•+ and R = 0.922 for DPPH). Total flavanol content was also highly correlated with TPC (R = 0.949) and antioxidant activities (R = 0.937 for DPPH, R = 0.979 for FRAP, and R = 0.997 for ABTS•+), while TSO showed no significant correlations with any of the examined parameters.

### 3.3. LC-MS Analysis of Carob Powders from Imera and Platses Cultivars

The workflow of Amerikanou et al. [28] was applied for a direct comparative characterization of phenolic profiles between two carob cultivars, thereby generating novel data on cultivar-specific phytochemical diversity. Tentative metabolite annotations were assigned when (a) the measured precursor ion *m*/*z* agreed with database values to three decimal places, and (b) at least two product-ion (MS/MS) fragments were concordant with reference spectra in library databases. Metaboanalyst 6.0 (https://www.metaboanalyst.ca/) was used for LC-MS/MS spectra processing and peak annotation. The spectra databases RIKEN (https://prime.psc.riken.jp/) and MSDIAL (https://systemsomicslab.github.io/compms/msdial/main.html (accessed on 9 July 2026)) was implemented for MS/MS peak annotation. Tentative identities and spectral characteristics of the compounds present in Imera and Platses carob extracts are reported in Table S1 (Supplementary Materials). From the 65 tentative carob metabolites identified in Amerikanou et al., 2025 [28], 35 of them are classified as phenolic compounds. The comparative study of their intensities in the two studied carob cultivars are presented in Table 3. The *p*-values reported for selected LC-MS features were generated by MetaboAnalyst using *t*-test as semiquantitative comparisons of relative peak intensities.

Assessing the findings of Table 3, the LC-MS analysis provided a preliminary semi-quantitative comparison of the phenolic fingerprints of Imera and Platses carob extracts. Overall, these results should be interpreted as preliminary due to the limited number of carob samples and sample replicates.

**Figure 1:**
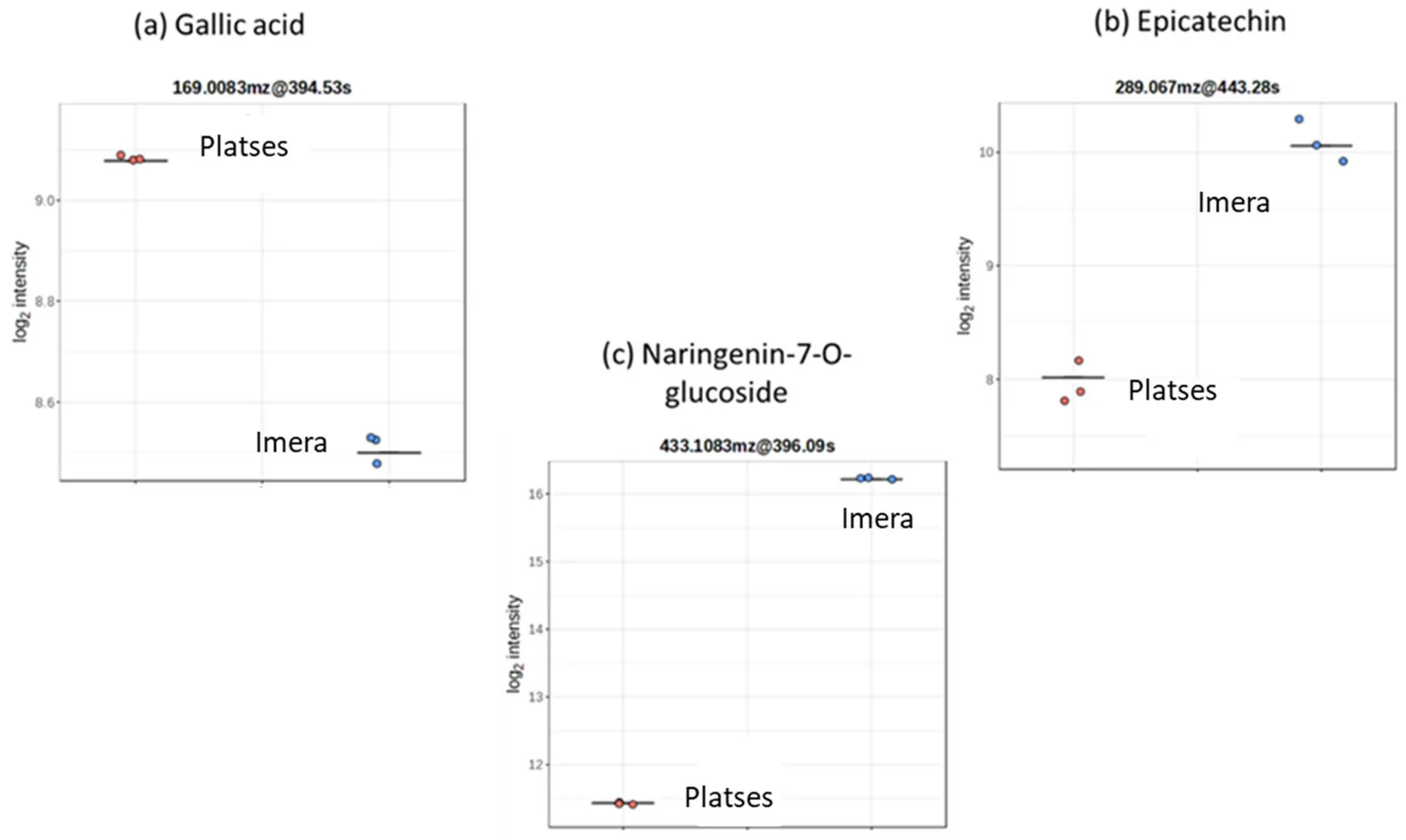
Box plots of (**a**) gallic acid; (**b**) epicatechin; (**c**) naringenin-7-O-glucoside for Platses and Imera cultivars (*n* = 3 run replicates for each sample).

In detail, phenolic profiling indicated cultivar-related trends in the relative abundance of the annotated compounds. Platses samples displayed higher intensities of phenolic acids (i.e., ferulic and gallic acid) (Figure 1a) and kaempferol. Imera tended to show increased intensities of flavonoids and galloylated derivatives, a finding that is consistent with its higher TPC and flavanol content (Table 2). Key flavanol derivatives, such as glycosides of kaempferol, epicatechin (Figure 1b) and important flavonoids, such as myricetin, catechin, apigenin, and naringenin glycosides (Figure 1c), showed increased intensities in Imera extracts, suggesting a greater contribution to antioxidant potential. Some compounds, such as 1,3,6-tri-O-galloylglucose and genistein, were detected in both cultivars, but intensities were higher in Imera, indicating a stronger presence of galloylated and isoflavonoid structures. Compounds such as ginnalin A, leucodelphinin, and taxifolin, which are recognized for their antioxidant properties [29], were also found in both cultivars, with alike intensities.

### 3.4. FT-IR Spectra Interpretation of Carob Extracts

**Figure 2:**
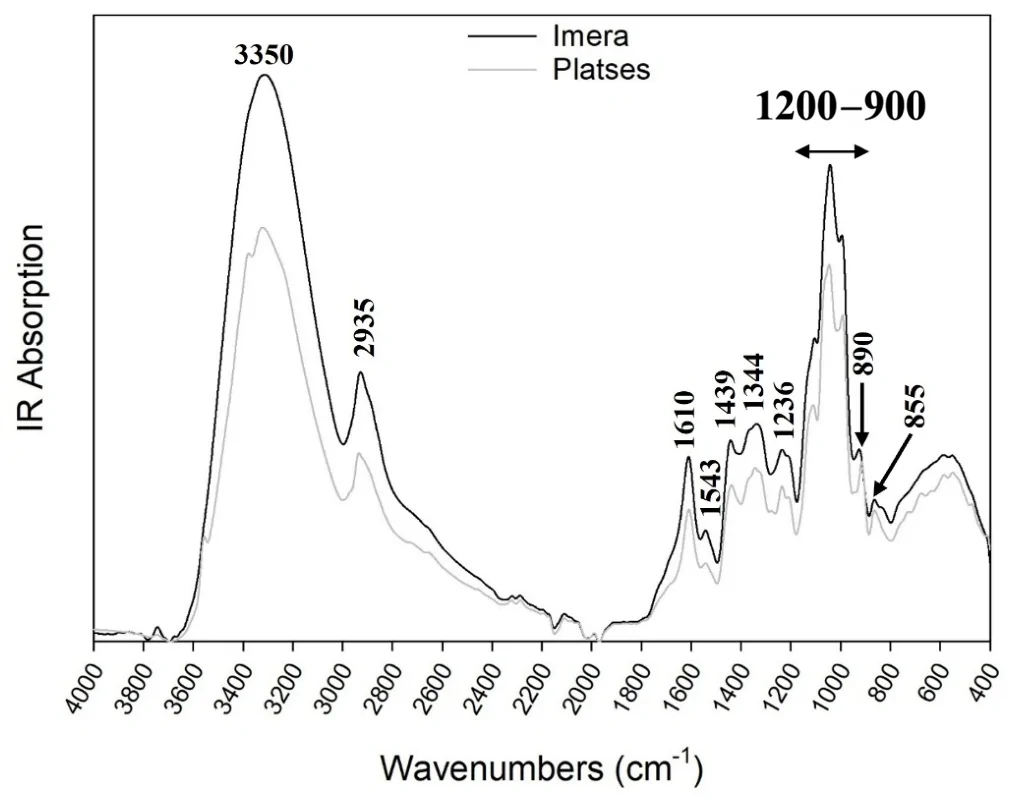
Recorded ATR-FTIR of carob powder samples from the Imera and Platses cultivars, with the main spectral regions of interest indicated. The broad band at 3350 cm^−1^ is mainly attributed to O–H stretching vibrations, while the band at 2935 cm^−1^ corresponds to C–H stretching. The region between 1200 and 900 cm^−1^ represents the carbohydrate fingerprint region, associated mainly with C–O and C–O–C stretching vibrations, with bands around 890 and 855 cm^−1^ indicating glycosidic linkages.

The observation of the recorded ATR-FTIR spectra of the samples allowed the detection of mostly quantitative differences between the samples in terms of peak IR absorption intensity (Figure 2). More specifically, the common peak around 3350 cm^−1^ can be assigned to the O-H and N-H stretching vibrations in phenolic compounds and carbohydrates, while the peak at 2935 cm^−1^ is associated with CH_2_ asymmetric stretching in aliphatic compounds. The peak at 1610 cm^−1^ can be attributed to the C=C and C=N stretching or the C=C-C stretching of aromatic ring bonds, while the peak at 1543 cm^−1^ is created due to the N-H deformation in proteins (Amide II band). The peaks at 1439 and 1344 cm^−1^ correspond to proteins (or lipids) and more specifically to the CH_2_ and CH_3_ bending vibrations respectively. The peak at 1236 cm^−1^ is associated with the C-O stretching vibration in esters, while the peaks between 1200 and 900 cm^−1^ are associated with the etheric bond vibration in sugars [21,30,31,32], while they assigned the peaks at 1210 and 1110 cm^−1^ to the C-O stretching (phenols) and C-H bending (fatty acids) respectively. In addition, they observed a correlation of peaks close to 3400 cm^−1^ to an increased number of phenolic hydroxyl groups in their samples. The findings in the present study (i.e., absorption intensities in the peaks at 3350, 1236, 1110 cm^−1^) could indicate an increased phenolic compound content in Imera cultivar. Regarding the content of polysaccharides in these two cultivars, Platses seems to be characterized by an increased absorption in the region corresponding to β-glycosidic linkages of pyranose compounds (i.e., peak around 890 cm^−1^) as well as in the region corresponding to α-glycosidic linkages of pyranose compounds (i.e., peak around 855 cm^−1^) [31], suggesting a potentially higher content in α- and β-glucans in Platses cultivar than in Imera cultivar.

### 3.5. Volatile Compounds

Retention times (RT, min) and relative abundances (%) of volatile compounds identified by GC-MS in the analyzed samples are given in Table 4 (Supplementary Materials). Isobutyric acid was the dominant compound across all samples, accounting for 43.36% in Imera and 40.67% in Platses, with no statistically significant difference observed between the two varieties (*p* = 0.149). Other short-chain fatty acids, including butyric acid (6.05% in Imera; 5.48% in Platses) and isovaleric acid (4.26% in Imera; 5.28% in Platses), were also highly abundant but displayed statistically comparable levels between genotypes (*p* > 0.05).

Despite the broad similarities in the major acid profiles, distinct and statistically significant varietal variations were established for specific volatile markers. A prominent differentiation was observed in the thermally induced furan derivatives. Specifically, tetrahydro-2,5-dimethyl-furan was significantly more pronounced in the Platses variety (6.00%) compared to Imera (2.44%) (*p* = 0.028). Additionally, the Imera variety exhibited a significantly higher relative abundance of tetradecanoic acid (3.51%) compared to Platses (1.07%) (*p* = 0.047). This statistical divergence highlights a stronger long-chain fatty acid and lipid-derived metabolic background for the Imera variety. Because samples were subjected to identical roasting parameters, these significant differences mark a potentially distinct pathway or reactivity during thermal processing of the pods from the two cultivars. Other lipid- and carbohydrate-derived volatiles, such as hexanoic acid, 2-heptanone, and various mid-to-long chain ketones (e.g., tridecyl methyl ketone and 6,10,14-trimethylpentadecan-2-one), contributed substantially to the overall profile of both varieties but did not yield statistically significant variations (*p* > 0.05) under the evaluated conditions.

## 4. Discussion

This is the first study to comprehensively characterize the phenolic composition, antioxidant capacity, FT-IR fingerprint and nutritional properties of edible carob powder from the underexplored Platses cultivar in comparison with the Imera cultivar.

Both cultivars showed comparable nutritional profiles with other carob cultivars of the Mediterranean region [33]. In our study, Imera exhibited higher protein and fat content, while Platses was richer in carbohydrates. The higher moisture content observed in Platses may be associated with favorable physicochemical properties, including lower rupture and bioyield forces, a lower modulus of elasticity, reduced rupture energy, and, consequently, improved processing efficiency [34].

While LC-MS identifies and quantifies specific, known compounds, it often underestimates the total phenolic content due to unknown or unseparated peaks. Combining both LC-MS and total phenolic cοntent assay allows researchers to cross-reference the total antioxidant capacity against individual chemical contributors. DPPH depicts the antioxidant and more specifically the free radical scavenging activity. Several DPPH activities have been reported for Carob methanolic extracts and usually range from 9.06 to 25.10 mg TE/g, depending on the country of origin and the grinding method [33,35]. Both Platses and Imera demonstrated relatively high DPPH activity, with Imera showing almost double values. This finding aligns with the other antioxidant assays tested herein, as Imera was also higher in TPC, FRAP, ABTS and flavanol content and showed enhanced ability to delay serum oxidization. Flavanols constitute a class of flavonoids that exert high radical scavenging activity due to their structure and their ability to easily give hydrogen atoms and terminate the flavonoidal aroxyl radicals [36]. In contrast, no statistically significant differences were observed in the TSO assay. This finding may be attributed to the different mechanism evaluated by the TSO assay, which reflects the inhibition of copper-induced serum lipid oxidation rather than direct radical scavenging or reducing capacity. Therefore, the lack of significant differences in TSO suggests that the higher antioxidant capacity observed by the chemical assays was not necessarily reflected in the inhibition of serum lipid oxidation under the experimental conditions. Finally, although increased resistance to serum oxidation was observed in Imera, TSO did not correlate with TPC, total flavanols and antioxidant assays, suggesting that higher phenolic content and antioxidant activity does not increase resistance to oxidation proportionally. This phenomenon is expected in biological systems, as antioxidants interact with serum endogenous compounds and increased or different compounds do not necessarily offer higher antioxidant protection [37].

Different genotypes may play important roles in the antioxidant power and in the phenolic makeup of Imera and Platses samples. Llompart et al. reported significant variabilities between distinct Balearic carob varieties. Cultivar-to-cultivar variability in the TPC, antioxidant and antiradical profiles, could be attributed to differences in the regulation of phenylpropanoid/flavonoid pathways, which change both the quantity and the presence or absence of phenolic compounds [38].

Ripening stage could play a role in the antioxidant capacity of carob powders, as phenolic compounds often reach a peak at different developmental stages. It is possible that distinct cultivars ripen differently and have different pulp-to-seed compositions, thus affecting TPC, FRAP and ABTS●+ values [39]. Focusing on Greek carob varieties, the intraspecific differences with regard to phenolic composition and antioxidant activity have been reported [23,40,41]. Cocoa powders have been reported as leading dietary sources of polyphenols with the highest total phenolic content (TPC) ranging from 9.2 to 57.4 mg GAE/g and antioxidant capacity values of 30.77–97.94 mg TE/g (DPPH), 73.97–267.43 mg TE/g (ABTS), and 28.88–98.74 mg TE/g (FRAP) [42]. Carob powder displayed significantly lower absolute TPC values in the Platses variety, while the Imera variety was significantly more elevated, reaching the lower bound of the reported TPC in cocoa powders. Furthermore, antioxidant activities of the Imera variety tested by DPPH and FRAP are close to the lower range of the antioxidant activity of cocoa powders. These results confirm that the antioxidant capacity of certain carob cultivars is considerable and that carob’s antioxidant activity may be comparable to that reported for cocoa, a polyphenol-rich food standard. Besides its antioxidant activity, carob is caffeine and theobromine free, has low lipid content and high fiber content. Due to these nutritional characteristics, carob may be considered appropriate for those who are sensitive to methylxanthines and also it can be used for the development of nutraceuticals and functional foods.

LC-MS analysis further supported these findings by revealing cultivar-dependent differences in phenolic composition. Specifically, the phenolic composition differed between Platses and Imera, indicating both qualitative and/or semi-quantitative variation in several metabolite classes, including phenolic acids, flavan-3-ols, flavonols, flavone glycosides, chalcone/dihydrochalcone derivatives, and tannin-related compounds. Such variation is consistent with recent evidence showing that different cultivars can differ in phenylpropanoid and tannin biosynthesis [23,43], and consequently in individual metabolites, such as gallic acid, ferulic acid, kaempferol (higher peak intensities in Platses cultivar), kaempferol glycosides, myricetin, apigenin and phloridzin derivatives (higher peak intensities in Imera cultivar). These differences are chemically plausible, since cultivar-dependent variation in the phenylpropanoid/flavonoid biosynthetic pathways can change both the overall abundance and the relative partitioning of metabolites into free phenolic acids, flavonoid aglycones, glycosylated flavonoids, and tannin-derived products. Recent work on different carob varieties cultivated under similar conditions has confirmed that genetic origin strongly influences antioxidant activity and phytochemicals composition, showcasing that at least part of the observed difference between Platses and Imera reflects cultivar-related metabolic differentiation [43]. The higher relative peak intensity of kaempferol glycosides, myricetin, and apigenin-related metabolites in Imera may be associated with its antioxidant potential and may reflect cultivar-dependent differences in flavonoid biosynthesis, especially glycosylation, which can differ substantially among cultivars. However, these comparative LC–MS data do not provide direct evidence of the contribution of individual metabolites to the measured antioxidant activity and therefore further targeted analyses are required to interrelated the concentration of specific metabolites with the antioxidant potential. Likewise, the stronger presence of phloridzin derivatives and other chalcone/dihydrochalcone-type metabolites in one cultivar may reflect differential regulation of branch pathways downstream of phenylpropanoid metabolism [21,38]. However, it should be noted that the present LC-MS study was designed as a comparative, semi-quantitative assessment and was based on a limited number of analytical replicates per cultivar and on relative peak intensities of tentatively annotated metabolites. Therefore, the observed differences should be interpreted as indicative cultivar-related trends and not as a global metabolomic discrimination of carob samples nor as evidence of absolute differences in metabolite concentrations, since such analysis would require a larger sample set and appropriate targeted quantitative validation. Furthermore, although both cultivars were roasted under identical conditions prior to analysis, thermal processing may have influenced the phenolic composition and volatile profile through the degradation of native compounds and the formation of Maillard reaction products. Therefore, while the observed differences likely reflect cultivar-dependent characteristics under the applied processing conditions, the contribution of roasting cannot be completely excluded.

FT-IR fingerprinting further supported the LC-MS findings. Imera exhibited more intense absorption bands in spectral regions associated with phenolics, i.e., the region around 3350 cm^−1^ and the region 1400–1200 cm^−1^ (more specifically at 1235 cm^−1^). Similarly, differences in polysaccharides, including alpha- (855 cm^−1^) and beta-glucans (890 cm^−1^), were evident with Platses displaying more intense absorption bands in spectral regions characteristic of these groups. These differences in composition stressed by FTIR support the importance of the appropriate cultivar selection for the production of antioxidant nutraceuticals or functional foods targeting gastrointestinal health.

The volatile composition of carob flours was shaped predominantly by varietal metabolic characteristics, with Imera and Platses exhibiting distinct biochemical signatures that determined their aromatic profiles. Across all samples, short-chain fatty acids represented the dominant class of volatiles, with isobutyric acid consistently emerging as the major constituent. This reflects the central role of branched-chain amino acid (BCAA) catabolism in plant tissues, where precursors like valine, leucine, and isoleucine undergo transamination and subsequent decarboxylation or dehydrogenation reactions that can give rise characteristic short-chain acids. Such amino-acid-derived pathways have been previously identified as key contributors to the aroma of carob-based products [9,10].

Clear varietal differentiation was evident in the abundance and distribution of targeted amino-acid-derived volatiles. Platses showed higher relative abundance of specific compounds linked to the turnover of branched-chain and aromatic systems, suggesting a potentially more active catabolism of precursor pools such as valine and phenylalanine, or a higher availability of these substrates as previously documented for specific carob genotypes. These differences may reflect varietal variation in the constitutive activity of aminotransferases and decarboxylases, which regulate the conversion of amino acids into aldehydes, acids, and related volatiles. Such genotype-dependent modulation of amino acid metabolism aligns with previous findings showing that carob varieties differ in their balance of amino-acid- and phenolic-derived pathways [44]. In contrast, Imera exhibited a stronger lipid-derived signature, characterized by significantly higher levels of long-chain saturated fatty acids, such as tetradecanoic acid. This pattern suggests potential varietal differences in lipid storage composition, *β*-oxidation dynamics, or the availability of fatty acid precursors [45]. The persistence of these lipid-derived volatiles across processing conditions suggests that lipid metabolism constitutes a stable varietal trait that continues to shape the aromatic outcome [9,46,47].

Varietal differences were also highly evident in compounds associated with thermally induced reactions, such as furans. The significantly more pronounced formation of tetrahydro-2,5-dimethylfuran in Platses (*p* ≤ 0.05) points to a higher abundance or thermal reactivity of sugar–amino acid conjugates, whereas the overall profile of Ιmera points to a comparatively stronger contribution of lipid-derived intermediates. These patterns likely reflect inherent varietal differences in the baseline pools of reducing sugars, free amino acids, phenolic compounds, and lipids—each of which channels thermal reactions toward distinct volatile outcomes [10,11].

Overall, the GC-MS data demonstrate that the aromatic identity of carob flour is driven primarily by varietal metabolic composition, with processing acting mainly as a modulator of pre-existing biochemical potentials. The central role of isobutyric acid, the varietal divergence in amino-acid- and lipid-derived volatiles (as exemplified by the significant differences in furanic compounds and tetradecanoic acid), collectively highlights genotype as the dominant determinant of carob aroma. These findings underscore the importance of varietal selection for tailoring the sensory and chemical properties of carob-based ingredients, particularly in applications where carob serves as a cocoa analog or a functional flavoring component.

The absence of quantitative data that lowers the strength of direct comparisons between Imera and Platses and the limited number of samples that constrains generalizability consist limitations of this study. Nevertheless, the work is strengthened by the use of multiple, complementary analytical methods across nutritional, antioxidant, chemical, and volatile profiles, providing a robust characterization and the major differences between pods of the Imera and Platses cultivars.

## 5. Conclusions

The present study demonstrated cultivar-dependent differences in the phenolic composition, antioxidant capacity, nutritional characteristics and volatile profiles of edible carob powders from the Imera and the Platses cultivars. The more pronounced phenolic acid profile observed in Platses suggests its potential application as a natural food preservative, consistent with the documented ability of gallic acid to enhance protection against lipid oxidation and microbial spoilage [48]. In contrast, the higher antioxidant activity, flavanol content and protein content of the Imera cultivar support its use in the development of antioxidant-rich functional foods and nutraceuticals. Overall, the observed cultivar-dependent differences highlight the importance of selecting the appropriate cultivar according to the intended application, while blending both cultivars may represent a promising strategy to combine enhanced antioxidant potential with improved preservative properties and nutritional quality.

## Figures and Tables

**Table 1:** Proximate composition of Imera and Platses carob pods.

Proximate Composition (%)	Platses Mean ± SD	Imera Mean ± SD
Moisture	9.20 ± 0.10 ^a^	6.60 ± 0.10 ^b^
Ash	2.53 ± 0.20	3.00 ± 0.03
Carbohydrates	48.83 ± 0.10 ^a^	49.07 ± 0.10 ^b^
Fat	0.24 ± 0.03 ^a^	0.33 ± 0.02 ^b^
Protein	6.10 ± 0.04 ^a^	7.40 ± 0.10 ^b^
Total dietary fibers	33.10 ± 0.40	33.60 ± 0.20

Different letters within the same row show statistically significant difference (*p* ≤ 0.05) between samples.

**Table 2:** Evaluation of phenolic content, flavonoid levels, and antioxidant activity of samples using various spectrophotometric assays.

Assessments	Platses Mean ± SD	Imera Mean ± SD
TPC (mg GAE/g dry matter)	4.0 ± 0.6 ^b^	9.0 ± 1.7 ^a^
Total flavanol content (μg catechin eq./g dry matter)	691.6 ± 37.1 ^b^	1919.7 ± 83.2 ^a^
DPPH radical scavenging activity (mg TE/g dry matter)	16.9 ± 1.7 ^b^	29.5 ± 3.7 ^a^
ABTS•+ (mg TE/g dry matter)	8.7 ± 0.3 ^b^	15.2 ± 0.3 ^a^
FRAP (mg Fe(II)/g dry matter)	39.2 ± 4.2 ^b^	80.0 ± 3.5 ^a^
TSO (sec)	4225.9 ± 263.8	5450.7 ± 1310.1

Different letters within the same row (method) show statistically significant difference (*p* ≤ 0.05) between samples. ABTS•+: 2,2′-azino-bis(3-ethylbenzothiazoline-6-sulfonic acid; DPPH: 2,2-diphenyl-1-picrylhydrazyl; FRAP: ferric reducing/antioxidant power, GAE: gallic acid equivalents; TE: Trolox equivalents, TPC: total phenolic content, TSO: total serum oxidizability assay (longer lag times indicate a higher level of antioxidant protection).

**Table 3:** Tentatively annotated phenolic compounds in the aqueous methanolic extracts of the *Imera* and *Platses* cultivars, as determined by LC-MS-TOF, and comparison of their relative peak intensities between the two cultivars.

Peak Number	Tentative Metabolite	Chemical Subclass (Based on HMDB Classification)	Cultivar (Highest Relative Intensity)
*Peak intensity ≥ 10* ^4^			
1	1,3,6-tri-O-galloylglucose	Tannins (Hydrolyzable tannins)	Imera *
2	Apigenin 6-C-arabinoside 8-C-glucoside (Isoschaftoside)	Flavonoid glycosides	Imera *
3	Apigenin 6-C-glucoside 8-C-arabinoside (Schaftoside)	Flavone Glycoside	Imera *
4	Catechin	Flavans	Imera *
5	Gallic acid	Benzoic acids and derivatives	Platses *
6	Genistein	Isoflav-2-enes	Imera *
7	Kaempferol	Flavones	Platses *
8	Methylgallate	Benzoic acids and derivatives	Platses *
9	Myricetin	Flavones	Imera *
*Peak intensity 10*^4^ − *10*^3^			
10	Astragalin (Kaempferol-3-glucoside)	Flavonol Glycoside	Imera *
11	Catechin gallate	Flavans	Imera *
12	Coumaroyl hexoside ^1^	Hydroxycinnamic acids and derivatives	No statistical difference between the two cultivars (*p*-value > 0.05)
13	Ellagic acid	Hydrolyzable tannins	No statistical difference between the two cultivars (*p*-value > 0.05)
14	Gallic acid hexoside ^1^	Benzoic acids and derivatives	Imera *
15	Genistin	Isoflavonoid Glycoside	Imera *
16	Ginnalin A	Benzoic acids and derivatives	No statistical difference between the two cultivars (*p*-value>0.05)
17	Isovitexin	Flavonoid Glycoside	Imera *
18	Luteolin-4′-O-glucoside	Flavonoid Glycoside	Imera *
19	Naringenin-7-O-glucoside	Flavonoid Glycoside	Imera *
20	Quercetin	Flavones	No statistical difference between the two cultivars (*p*-value > 0.05)
*Peak intensity ≤ 10* ^3^			
21	2′,4′,6′-Trihydroxydihydrochalcone	Chalcones and dihydrochalcones	Imera *
22	2′,6-Dihydroxyflavone	Flavones	Platses *
23	Benzoic acid	Benzoic acids and derivatives	No statistical difference between the two cultivars (*p*-value > 0.05)
24	Cinnamic acid	Cinnamic acids	Imera *
25	Epicatechin	Flavans	Imera *
26	Epigallocatechin	Flavans	Platses *
27	Esculetin	Hydroxycoumarins	Platses *
28	Ferulic Acid	Hydroxycinnamic acids and derivatives	Platses *
29	Kaempferol-3-Glucoside-2″-p-coumaroyl	Flavonoid glycosides	Imera (ND in Platses)
30	Leucodelphinidin	Flavans	No statistical difference between the two cultivars (*p*-value > 0.05)
31	Liquiritin	Flavonoid Glycoside	Imera *
32	Phloridzin derivative	Flavonoid Glycoside	Imera *
33	Secoisolariciresinol	Dibenzylbutanediol lignans	Platses *
34	Taxifolin	Flavans	No statistical difference between the two cultivars (*p*-value > 0.05)
35	Tricetin	Flavones	No statistical difference between the two cultivars (*p*-value > 0.05)

ND: not detected; *: indicates statistically significant difference between cultivars (*p*-value ≤ 0.05); ^1^: these metabolites were tentatively annotated as sugar-containing phenolic compounds; therefore, their definitive chemical classification may vary depending on the nature and position of the linkage between the phenolic moiety and the hexose, once their structures are fully elucidated.

**Table 4:** Volatile compounds identified by GC-MS in carob samples from the Imera and Platses cultivars.

Compound	Imera Mean ± SD	Platses Mean ± SD
Furan, tetrahydro-2,5-dimethyl-	2.44 ± 0.31 ^a^	6.00 ± 1.47 ^b^
Ethane, 1,1-diethoxy-	3.01 ± 1.83	3.80 ± 1.12
Isobutyric acid ethyl ester	0.22 ± 0.15	1.34 ± 1.67
2-Furanmethanol, tetrahydro-	0.58 ± 0.04	0.67 ± 0.25
Butyric acid ethyl ester	0.34 ± 0.08	0.16 ± 0.08
Isobutyric acid	43.36 ± 1.58	40.67 ± 2.16
Butyric acid	6.05 ± 0.08	5.48 ± 0.72
Isovaleric acid	4.27 ± 0.54	5.28 ± 0.57
Heptan-2-one/2-Heptanone	1.38 ± 0.14	0.46 ± 0.24
Hexanoic acid, ethyl ester	2.01 ± 0.37	1.56 ± 0.15
Hexanoic acid	12.53 ± 0.74	12.29 ± 2.70
2-Nonanone	1.57 ± 0.70	1.41 ± 0.11
Nonanal	0.34 ± 0.05	0.68 ± 0.17
Octanoic acid	3.02 ± 0.93	2.91 ± 2.09
Nonanoic acid	2.16 ± 0.94	1.90 ± 1.22
Decanoic acid	0.84 ± 0.32	0.62 ± 0.09
Phenol, 2,4-bis(1,1-dimethylethyl)-	1.53 ± 0.41	1.98 ± 0.22
(Z)-Pentadec-6-en-2-one	0.86 ± 0.79	1.85 ± 0.78
Tridecyl methyl ketone	1.40 ± 1.10	3.01 ± 1.38
Tetradecanoic acid	3.51 ± 0.80 ^a^	1.07 ± 0.24 ^b^
6,10,14-trimethylpentadecan-2-one (Phytone)	4.35 ± 1.93	5.02 ± 1.37
Tetradec-(7Z)-en-2-one	3.13 ± 0.51	2.56 ± 0.20

The percentages represent the relative abundance among the identified peaks. Results are presented as mean values ± standard deviation (SD). Different letters indicate statistically significant difference (*p* ≤ 0.05) between samples.

## Data Availability

Data will be made available on request.

## Supplementary Materials

The following supporting information can be downloaded at https://www.mdpi.com/article/10.3390/foods15183197/s1, Table S1: Tentatively annotated metabolites identified by LC-TOF-MS profiling of carob extracts. Table S2: Retention times of volatile compounds identified by GC-MS.

## Abbreviations

The following abbreviations are used in this manuscript:

ABTS	2,2′-Azino-bis(3-ethylbenzothiazoline-6-sulfonic acid) radical assay
AOAC	Association of Official Analytical Chemists
ATR	Attenuated Total Reflectance
BCAA	Branched-Chain Amino Acids
DMACA	p-Dimethylaminocinnamaldehyde
DPPH	2,2-Diphenyl-1-picrylhydrazyl
ESI	Electrospray Ionization
FRAP	Ferric Reducing Antioxidant Power
FT-IR/FTIR	Fourier Transform Infrared Spectroscopy
GAE	Gallic Acid Equivalents
GC-MS	Gas Chromatography–Mass Spectrometry
IDA	Information Dependent Acquisition
LC-MS	Liquid Chromatography–Mass Spectrometry
LC-MS/MS	Liquid Chromatography Tandem Mass Spectrometry
LC-TOF-MS	Liquid Chromatography–Time-of-Flight Mass Spectrometry
MS	Mass Spectrometry
MS/MS	Tandem Mass Spectrometry
PBS	Phosphate-Buffered Saline
RT	Retention Time
SD	Standard Deviation
TE	Trolox Equivalents
TPC	Total Phenolic Content
TSO	Total Serum Oxidizability assay
