# Supplementary material for: Antioxidant Capacity and Phenolic Profile of Edible Carob Pods (Ceratonia siliqua L.)
Source: Foods. 2026 Sep 10;15(18):3197. doi: 10.3390/foods15183197 (PMC13605526; doi:10.3390/foods15183197)
Supplement: Supplementary file 1 [file foods-15-03197-s001.zip › foods-4460819-supplementary.pdf]

Table S1. Annotated putative carob metabolites obtained by LC-TTOF MS profiling.

Peak number	Tentative metabolite	Chemical Subclass (based on HMDB classification)	m/z (MS/MS2)	RT (min)
	Peak intensity ≥10^4			
1	Apigenin 6-C-arabinoside 8-C-glucoside (Isoschaftoside)	Flavonoid glycosides	563.1677/473.1001 443.1166 193.0496	6.22
2	Catechin	Flavans	289.0700/245.0080 151.0419 137.0247 125.0249 109.0295	5.9
3	Myricetin	Flavones	317.0262/271.0098 178.9989 151.0026 137.0243	7.29
4	Apigenin 6-C-glucoside 8-C-arabinoside (Schaftoside)	Flavone Glycoside	563.1510/545.1266 503.1127 473.1060 443.0960 425.0801 383.0747 353.0644	6.12
5	Kaempferol	Flavones	285.0447/255.0290 240.0399 227.0350 117.0346	7.51
6	1,3,6-tri-O-galloylglucose	Tannins (Hydrolyzable tannins)	635.1101/483.0706 465.0610 313.0532 169.0139	6.16
7	Genistein	Isoflav-2-enes	269.0619/241.0536 224.0449 213.0540 201.0559 182.0462 173.0403 169.0610 133.0295 117.0324 91.0192	7.52
8	Methylgallate	Benzoic acids and derivatives	183.0077/140.0117 139.0036 136.9873	6.35
9	Gallic acid	Benzoic acids and derivatives	168.9967/125.0234 124.0151 123.0121	4.32
	Peak intensity 10^4			
10	Catechin Gallate	Flavans	441.1381/149.0452, 125.0245	5.92
11	Naringenin-7-O-glucoside	Flavonoid Glycoside	433.1197/313.0658, 271.0610, 151.0038	6.64
12	Luteolin-4'-O-glucoside	Flavonoid Glycoside	447.1060/341.0879,	6.75

			123.0446	
13	Isovitexin	Flavonoid Glycoside	431.0983	6.98
14	Quercetin	Flavones	301.0178/273.0426, 178.9973, 161.0246, 151.0042, 121.0030, 109.0298, 107.0140	7.58
15	Astragalin (Kaempferol-3-glucoside)	Flavonol Glycoside	447.1060/285.0853, 225.0437	6.75
16	Genistin	Isoflavonoid Glycoside	431.0983/89.0221, 87.0752	6.98
17	Gallic acid hexoside	Benzoic acids and derivatives	331.0213/169.0130, 125.0239	4.15
18	Coumaroyl Hexoside	Hydroxycinnamic acids and derivatives	325.1000/145.0302, 119.0497	6.06
19	Ellagic Acid	Hydrolyzable tannins	300.9964/283.9966, 185.0247	6.62
20	Ginnalin A	Benzoic acids and derivatives	467.2121/301.0042, 169.0191	7.3
	Peak intensity 10^3			
21	Leucodelphinidin	Flavans	305.0637/221.0310, 137.0230, 125.0240	6.06
22	Phloridzin derivative	Flavonoid Glycoside	465.1272/151.0378	6.53
23	Esculetin	Hydroxycoumarins	177.0113/132.0168, 121.0331, 107.0434, 104.0262	6.88
24	2',4',6'-Trihydroxydihydrochalcone	Chalcones and dihydrochalcones	257.1328/122.0377, 109.0307, 93.0333	8.25
25	Epicatechin	Flavans	289.0647/221.0775, 205.0425, 203.0646, 149.0222, 125.0256, 123.0459	6.17
26	Epigallocatechin	Flavans	305.0732/144.0479, 125.0399	6.36
27	Taxifolin	Flavans	303.0298/199.0378, 177.0176, 151.0042, 125.0236, 109.0274	7.07

28	2',6-Dihydroxyflavone	Flavones	252.9774/223.0432, 195.0449, 167.0477, 133.0290, 117.0345	7.5
29	Tricetin	Flavones	301.0852/191.0337, 151.0040, 149.0243	7.35
30	Liquiritin	Flavonoid Glycoside	417.0985/255.0652, 135.0089, 119.0508	6.7
31	Kaempferol-3-Glucoside-2''-p-coumaroyl	Flavonoid glycosides	593.1322	5.93
32	Secoisolariciresinol	Dibenzylbutanediol lignans	407.1456/361.1603, 346.1376, 165.0546	6.61
33	Ferulic Acid	Hydroxycinnamic acids and derivatives	193.0448/134.0381, 133.0288	6.92
34	Cinnamic acid	Cinnamic acids	147.0439/101.0395	7.86
35	Benzoic acid	Benzoic acids and derivatives	121.0298/92.0337	7.22

Table S2. Retention times (RT, min) of volatile compounds identified by gas chromatography–mass spectrometry (GC-MS) in the analyzed samples. Compounds are listed according to their elution order on the chromatographic column. Identification was achieved by comparison of mass spectra with reference library databases.

**Volatile compounds**	**R.T**
Furan, tetrahydro-2,5-dimethyl-	2.117
Ethane, 1,1-diethoxy-	2.588
Isobutyric acid Ethyl-ester	3.419
2-Furanmethanol, tetrahydro-	3.713
Butyric acid Ethyl-ester	4.659
Isobutyric acid	6.089
Butyric acid	6.325
Isovaleric acid	6.911
Heptan-2-one	7.515
Hexanoic acid, ethyl ester	11.145
Hexanoic acid	13.044
2-Nonanone	14.221
Nonanal	14.673
Octanoic acid	17.684
Nonanoic acid	19.964
Decanoic acid	22.653
Phenol, 2,4-bis(1,1-dimethylethyl)-	26.303
(Z)-Pentadec-6-en-2-one	30.339
Tridecyl methyl ketone	31.009
Tetradecanoic acid	32.504
6,10,14-trimethylpentadecan-2-one (hexahydrofarnesylacetone or Phytone)	34.199
Tetradec-(7Z)-en-2-one	34.965
